# Diagnosis of *Plasmodium* infections using artificial intelligence techniques versus standard microscopy in a reference laboratory

**DOI:** 10.1128/jcm.00775-24

**Published:** 2024-12-10

**Authors:** Sanjai Nagendra, Roxanna Hayes, Dayeong Bae, Krystin Dodge

**Affiliations:** 1 LabCorp 687747 , Burlington, North Carolina, USA; 2 Noul Co., Ltd. 663275 , Yongin, Republic of Korea; Mayo Clinic Minnesota, Rochester, Minnesota, USA

**Keywords:** *Plasmodium*, diagnosis, artificial intelligence

## Abstract

Diagnosing malaria using standard techniques is time-consuming. With limited staffing in many laboratories, this may lead to delays in reporting. Innovative technologies are changing the diagnostic landscape and may help alleviate staffing shortages. The miLab MAL, an automated artificial intelligence-driven instrument was compared with standard microscopy at LabCorp reference laboratories. Four hundred eight samples submitted for parasitic examination were prepared with thick and thin smears and Noul’s malaria platform miLab MAL, and evaluated for positivity, negativity, percent positivity, and species identification. Of 408 samples, 399 samples were manually negative, while 397 were negative by miLab MAL. Two samples initially classified as negative manually were found positive by miLab MAL. In all nine cases, *Plasmodium falciparum* was identified by both methods. Percentage parasitemia was higher in the manually calculated method, especially when >1%. miLab MAL was accurate in identifying the absence of *Plasmodium falciparum* and exhibited higher sensitivity than the manual method. All positive samples detected by microscopy were also identified with miLab MAL. All positive *Plasmodium* cases were correctly identified by miLab MAL. However, the number of positive samples was limited to only *Plasmodium falciparum*. Although parasitemia by the manual method was on average six times higher than with miLab MAL, this may be due to sampling variability. The findings show that miLab MAL can be used to screen out negative *Plasmodium falciparum* samples. Further studies assessing parasitemia between methods and identification of non-falciparum samples are necessary to assess the reliability of this new technology.

## INTRODUCTION

Affecting more than 247 million people and causing 625,000 deaths worldwide, malaria is a major global cause of morbidity and mortality (1). With ever-increasing global travel, imported cases of malaria are increasingly appearing in non-endemic countries. In 2018, 1,823 imported cases of malaria were identified in the United States, the highest number of cases there since 1972 (2). In addition, locally transmitted malaria cases were reported in Florida, Maryland, and Texas in 2023 for the first time in 20 years (3, 4). As air travel increases post-pandemic, imported malaria cases in non-endemic countries may continue to rise.

Laboratory diagnosis of malaria in non-endemic countries using standard thin and thick smears is challenging. Although the number of imported malaria cases is increasing, most laboratories see only a few cases yearly. In addition to the low volume of cases seen, there is variability between laboratories regarding smear preparation, staining, and interpretation. Discrepancies and delays in diagnosis can occur (5, 6). The landscape of parasite diagnosis is transforming with the rapid evolution of artificial intelligence (AI)-driven instrumentation. This paradigm shift is particularly evident in deploying innovative technologies such as the miLab MAL, an automated AI-driven instrument. This paper examines the miLab MAL, designed for use in endemic areas and non-endemic health facilities. The miLab platform utilizes hydrogel stamping technology, staining, and immunostaining to automatically generate liquid-free stained blood smears. These smears are the foundation for a diagnostic process facilitated by a digital microscope featuring one 50× objective lens used under dry conditions without an adjustable aperture and a complementary metal oxide semiconductor sensor. Subsequently, corresponding cell images are acquired, and an AI algorithm is employed to analyze these images, enabling the quantification and identification of malaria parasites.

A comparative study was conducted in a high-volume reference laboratory to assess the efficacy of this diagnostic approach. The study aimed to juxtapose malaria detection through conventional microscopy with the miLab MAL to understand whether new technologies can increase accuracy and efficiency in malaria diagnosis.

## MATERIALS AND METHODS

Four hundred nine K_2_ EDTA whole blood samples submitted for parasitic examination from North Carolina, South Carolina, Virginia, the District of Columbia, and Maryland between August 2023 and September 2023 were prepared with traditional methods (thick and thin smears) and Noul’s malaria AI-driven instrument, miLab MAL.

Thin and thick blood smears were prepared and stained with Wright-Giemsa stain. After evaluation, thick smears were scanned at low power (10×) and 100 oil immersion fields (100×) were examined, looking for intracellular and extracellular parasites. Then, 300 oil immersion fields of the thin film were examined for intracellular and extracellular parasites. The medical technologist reported the samples as negative if parasites were not seen. If parasites were seen, a percentage of parasitemia was calculated by counting the number of parasitized RBCs seen per 1,000 RBCs. In addition, the medical technologist reviewed the smear for red cell inclusions which could potentially interfere with miLab MAL interpretations. All positive cases of parasites were referred to a hematopathologist for interpretation. After reviewing positive cases, the hematopathologist identified the species of the parasites and noted the percentage of malarial forms present. Turnaround time for reporting samples was between 12 and 24 hours for negative samples and greater than 24 hours for positive samples needing pathologist interpretation.

**Fig 1:**
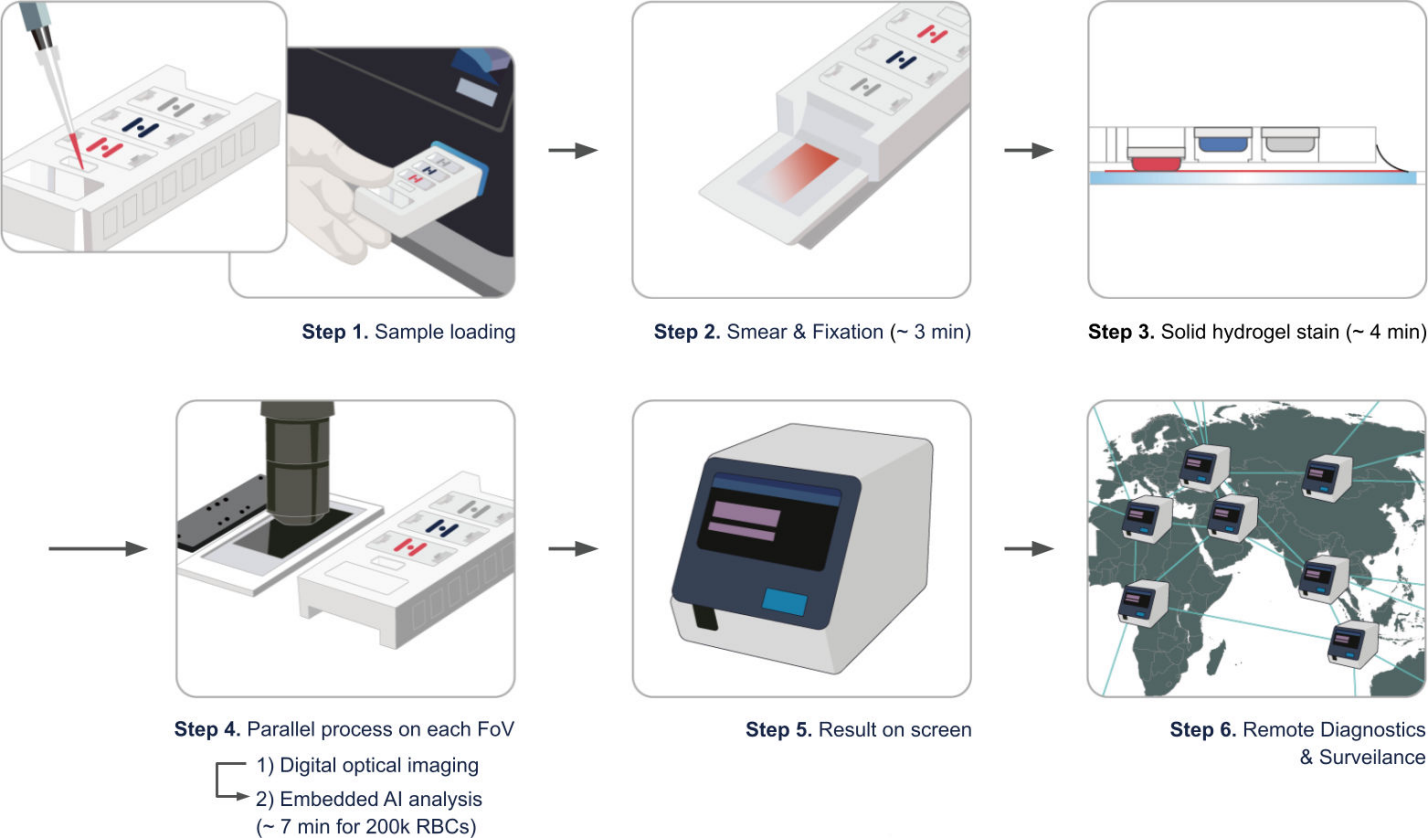
Schematic of the miLab MAL workflow. Step 1: a blood sample is loaded into the cartridge. Once the blood-loaded cartridge is positioned into the input slot, it is automatically inserted into the miLab platform. Step 2: blood is smeared by the polymer film in the cartridge, and the smeared blood is fixed. Step 3: the target area of the smeared and fixed sample is stained by stamping using hydrogel patches. The eosin, methylene blue, and buffer-containing patches are stamped in order. Step 4: digital optical images are processed for each FoV, and AI analysis proceeds in parallel. Step 5: the result is shown on the screen. The user can confirm the result. Step 6: the miLab platform can be connected to the internet if required. Remote diagnostics and surveillance can be performed using web-based software, miLab Viewer, providing all raw data and AI analysis results with patient information. The time to analyze from Step 1 to Step 6 is 18 minutes with Step 6 (interpretation performed within 3 minutes).

For the miLab MAL, deidentified whole blood samples in K_2_ EDTA tubes were homogenously mixed and inverted 10 times. Using a micropipette, 5 µL of whole blood was loaded into the miLab malaria cartridge. After blood was smeared by the polymer film in the cartridge, the smear was fixed. The fixed sample was stained with hydrogel patches containing eosin, methylene blue, and buffer. Two hundred thousand erythrocytes were chosen for screening. Using AI technology, cropped images suspicious of *Plasmodium* were displayed on the screen for the Noul medical laboratory technologist to review remotely from the Republic of Korea. Approximately 400–600 field of views (FoV) were captured with an individual pixel size of 0.15 mm × 0.15 mm and an image resolution of 1,920 × 1,200 pixels. Images were available off the device in JPEG format. The Noul medical laboratory technologist confirmed that the indicated images were positive or negative for parasites. If the sample was positive, the species was identified by miLab MAL and confirmed by the technologist. Processing and analysis of the miLab specimen took an average of 18 minutes including 7–8 minutes for processing, 7–8 minutes for imaging and AI analysis, and 3 minutes for interpretation by the technologist. The schematic of the miLab MAL workflow is shown in Fig. 1. The percent parasitemia was calculated using the following formula after the miLab operator reviewed and confirmed the result analyzed by miLab MAL:


$$Parasitemia\;\left(\%\right)=\frac{Number\;of\;Plasmodium\;forms\;\left(trophozoites\;and\;schizonts\;\right)}{\;number\;of\;counted\;erythrocytes}\times \;100$$

Both the Noul team, medical technologist, and pathologist performing standard microscopy were blinded to each other’s results. Correlation of methods was performed by the pathologist after all results were interpreted.

## RESULTS

**Fig 2:**
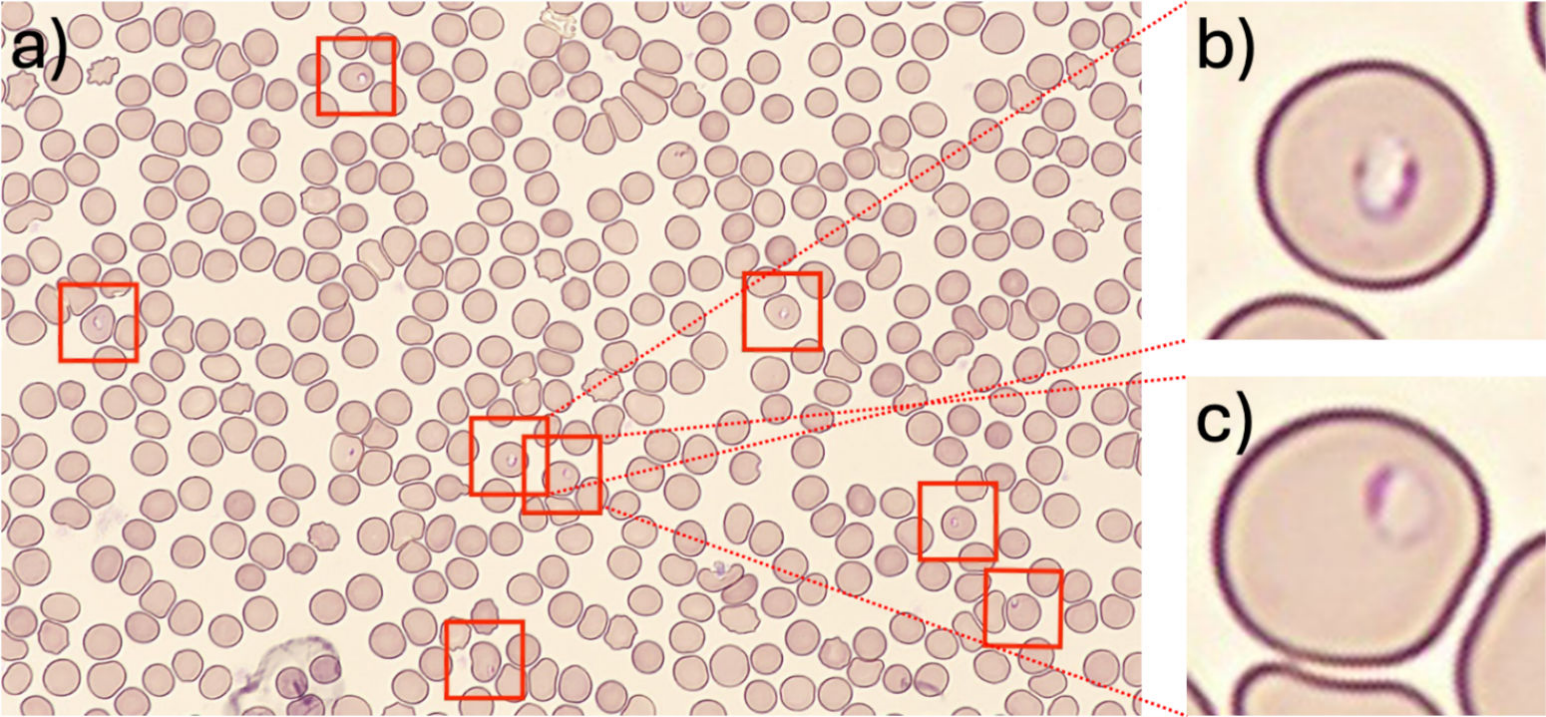
The miLab MAL images of case No. 7 sample. (a) miLab MAL field image is shown on the result screen from case 7, the *P. falciparum* positive sample. (**b** and **c**) One of the cell images, miLab MAL showed as a suspicious cell on the result screen.

**Fig 3:**
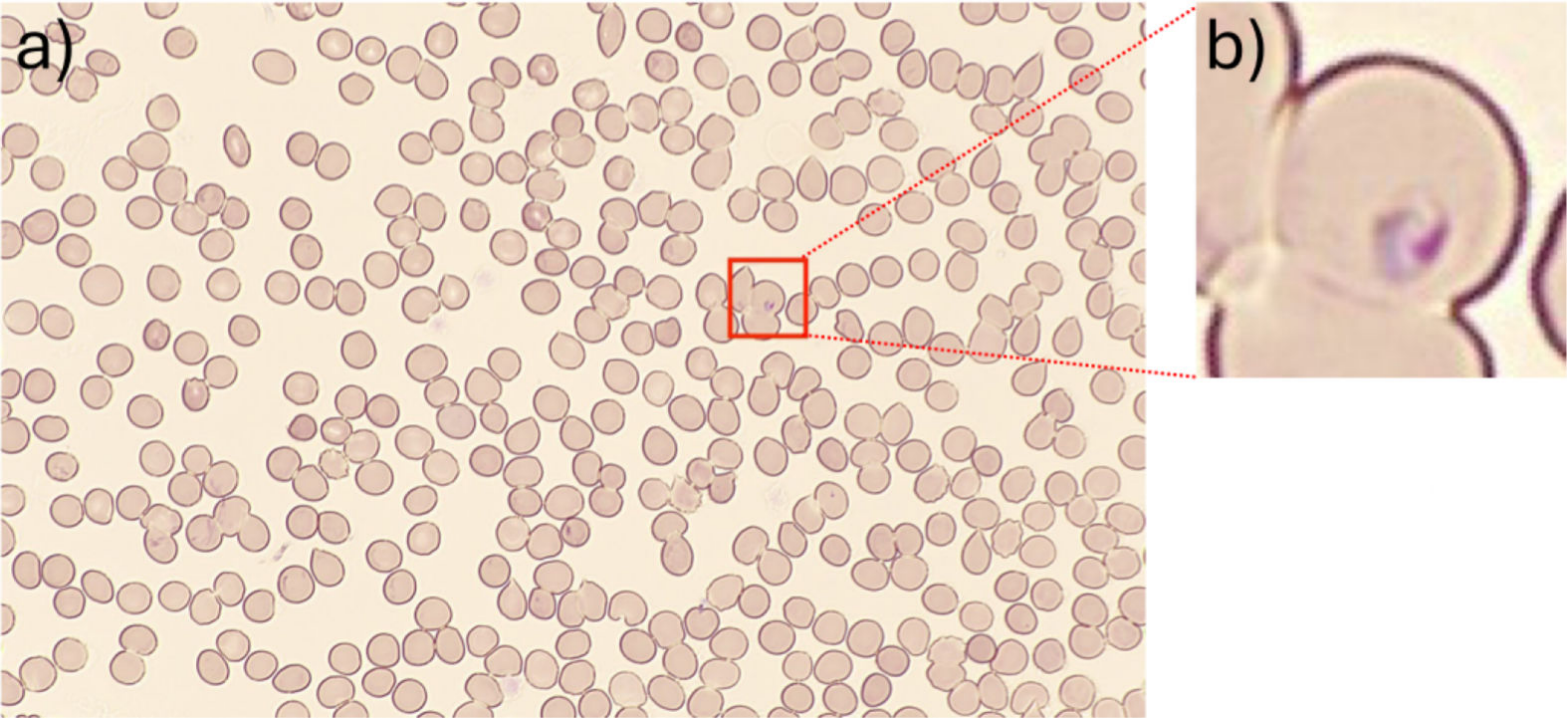
The miLab MAL images of case No. 8 sample. (**a**) miLab MAL field image is shown on the result screen from case 8, the *P. falciparum* positive sample, initially classified as negative by the manual method. (**b**) One of the cell images, miLab MAL showed as a suspicious cell on the result screen.

This study initially examined 409 samples; one sample was interpreted as *Babesia* species on microscopy and excluded from the study. In the remaining 408 samples, the standard microscopic method identified 399 samples as negative and 9 as positive for *Plasmodium* species. Utilizing miLab MAL, 397 samples were classified as negative, while 11 were identified as positive. Sensitivity, specificity, positive predictive value (PPV), and negative predictive value (NPV) were all 100% for the miLab MAL whereas sensitivity, specificity, PPV, and NPV were 81.8%, 100%, 100%, and 99.5%, respectively, for the traditional microscopic method. Table 1 summarizes the comparative analysis between the traditional microscopic method and the miLab MAL in detecting *Plasmodium* species. Table 2 highlights the diagnostic testing accuracy of both methods. The field image and cell images that miLab MAL identified as positive and *Plasmodium falciparum* are shown in Fig. 2. All 9 samples identified as positive by the standard microscopic method were concurrently positive when assessed using the miLab MAL. Two samples initially classified as negative by the standard microscopic method were found positive by miLab MAL. Upon manual re-examination of thin and thick smears, the pathologist found very rare trophozoites, constituting less than 0.1% of the erythrocytes in both samples. After the miLab MAL images noted in Fig. 3 were correlated with rare trophozoites noted on the smear, the discrepancy was reconciled and a diagnosis of low parasitemia *P. falciparum* infection was confirmed.

**TABLE 1:** Comparison of the malaria diagnostic results from traditional microscopic methods and from the miLab MAL analysis: 408 K_2_ EDTA whole blood samples submitted for parasitic examination from August and September 2023^*a*^

Comparison (*n* = 408)		Traditional microscopic method		
		Positive	Negative	Total
miLab MAL analysis	Positive	9	2	11
	Negative	0	397	397
	Total	9	399	408

^*a*^ One case of *Babesia* was excluded from the study.

**TABLE 2:** Diagnostic accuracy of traditional microscopy and miLab MAL

	Sensitivity (%)	Specificity (%)	PPV (%)	NPV (%)
Traditional microscopy	81.8	100	100	99.5
miLab MAL	100	100	100	100

Among the 11 positive smears identified by both methods, parasitemia quantitation was feasible in 9 cases using the standard microscopic method and the miLab MAL. In two positive microscopy cases, parasitemia calculation was not possible. In the first instance, cellular degeneration hindered the microscopic assessment of parasitemia, while in the second case, the parasitemia value was not reported.

Comparing parasitemia values between the two methods revealed higher parasitemia counts by standard microscopy. Parasitemia percentages on average were six times higher with standard microscopy than with the miLab MAL (range 0.5–27 times greater), especially when parasitemia was greater than 1% by standard microscopy.

In 9 of 11 positive cases, species identification was available with both methods. In 9 of 9 cases where species identification was performed, *P. falciparum* was identified by both methods.

Red cell inclusions were identified in 2/408 samples (one case each of basophilic stippling and Howell-Jolly bodies). The miLab MAL did not inadvertently identify these inclusions as malarial parasites and therefore did not cause a false positive result.

## DISCUSSION

Microscopic examination of thin and thick smears is used worldwide to diagnose malaria and is considered the “gold standard.” When low levels of parasitemia are suspected, thick smears are performed to improve sensitivity by more than 11 times (7, 8). Although less sensitive, thin smears have multiple advantages; they are not only helpful in morphological analysis and screening for parasites when no thick smear is available (9) but also aid in parasitemia calculations. Interpretation of these malaria smears is time-consuming and requires extensive training of medical laboratory professionals. The United States is estimated to have a shortage of 25,000 medical laboratory technologists (10), making it exceedingly difficult to find adequately trained professionals for malaria interpretation. Technological advancements in AI-driven technologies are beginning to transform laboratory medicine and potentially alleviate workforce shortages.

Computer-assisted diagnosis of malaria based on machine-learning algorithms is rapidly being introduced to improve timely and accurate malaria diagnoses (11). Several such diagnostic platforms have been tested. An automatic image analysis identification system analyzing thick blood films correctly classified *P. falciparum* and *P. vivax* in 95% of parasite-positive films (12). Machine learning models based on a convolutional neural network have accurately classified *Plasmodium* species on thin smears 97% of the time (13). Image processing and AI techniques developed on mobile phones and tablets have also accurately assessed malaria-positive samples with 91% accuracy (14). Unlike the other technologies mentioned above, the miLab technology integrates all preparation, imaging, and AI analysis processes. The miLab MAL had a sensitivity of 94.3% and a specificity of 94.0% for *P. falciparum* from a previous study and is expected to equal or exceed a WHO-qualified level 1 microscopist (achieving 90% or better accuracy for species detection and identification and >50% accuracy in parasite count) (15).

In our side-by-side comparison, testing with the miLab MAL was accurate in identifying intraerythrocytic parasites and exhibited higher sensitivity than the traditional method. All the positive samples detected in microscopy were also identified with the miLab MAL. The miLab MAL also detected *Plasmodium* ring forms in two additional cases, initially negative by microscopy. Parasitemia levels were lower than 0.1% in these two cases.

The miLab MAL’s improved sensitivity in detecting *Plasmodium* species compared to traditional methods was not surprising. Studies have shown that microscopy may miss more than 25% of malaria cases, especially in areas of low malaria transmission (16). Therefore, AI technologies such as miLab MAL will help improve diagnostic accuracy, especially in low-parasitemia settings (17).

All cases negative for *Plasmodium* species by microscopy were also negative by the miLab MAL method (100% specificity). Although a limited number of intraerythrocytic inclusions were seen in this study (*n* = 2), miLab MAL did not confuse Howell-Jolly bodies or basophilic stippling with parasites. Additional studies with a greater number of red cell inclusions may be helpful in ascertaining how inclusions affect miLab MAL interpretation.

**Fig 4:**
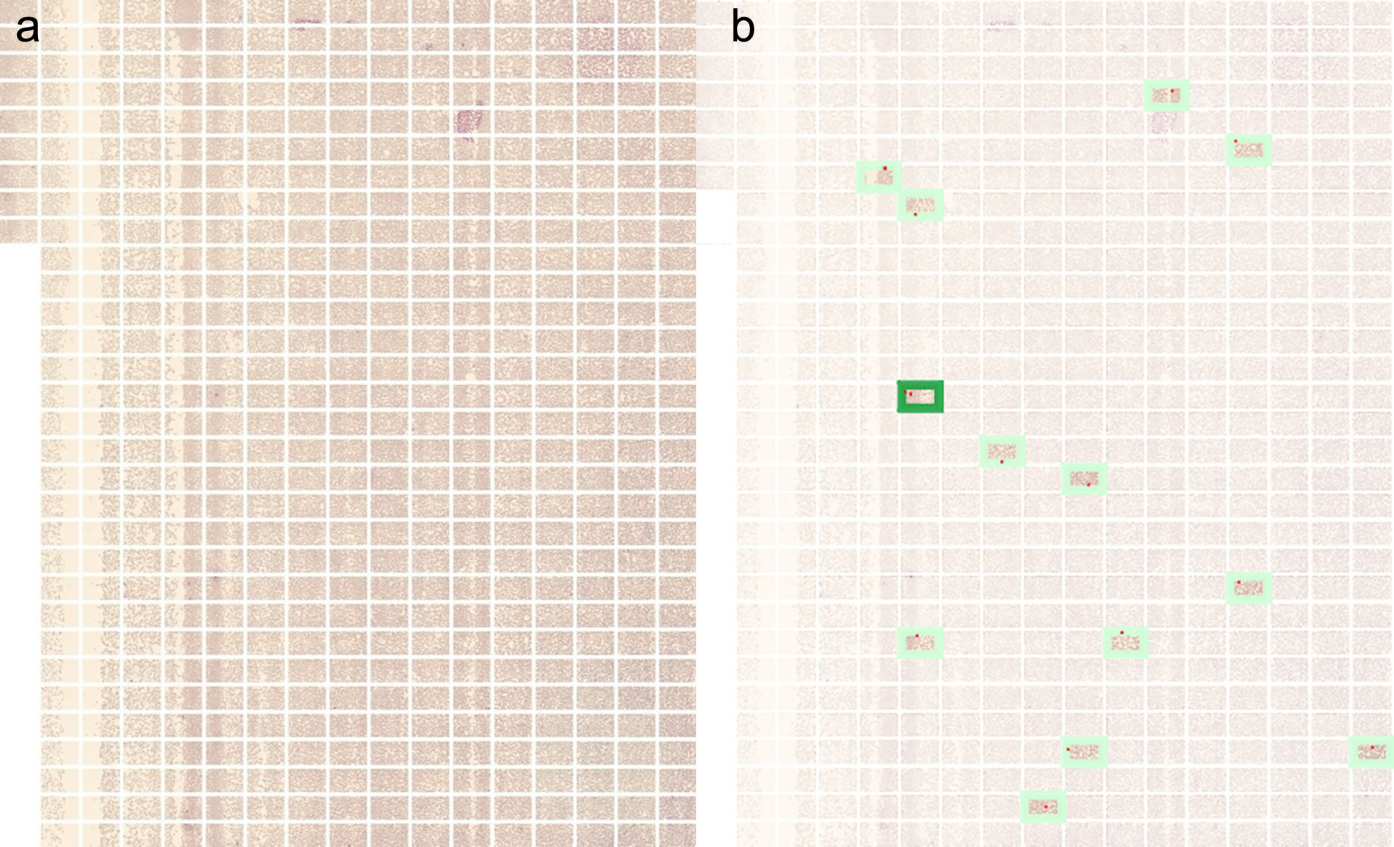
(**a**) Image of the stained area of the miLab slide. (**b**) Fields with a green box showing parasitized erythrocytes.

The cause of the differences in parasite counts between methods is uncertain. A few factors suggest that miLab MAL is more accurate in detecting parasitemia as it counts 200,000 erythrocytes compared to the 1,000 erythrocytes counted by standard microscopy. Differences can occur because parasite density varies across the smeared areas of the blood film, with miLab counting 200 times more erythrocytes in a given area. The technologist who counts parasite density may also inadvertently overestimate parasitemia by favoring counting microscopic fields with visible parasites. This phenomenon, known as Poisson variability, has been previously described in the literature (18). The urgency of addressing this issue in related research is underscored by the composite miLab MAL-stained images showing the variability of parasitized erythrocytes in a smear, as noted in Fig. 4. A prior study (15) found that the miLab method exhibited an approximate twofold systematic underestimation of parasite density.

Even when microscopic examinations are performed by highly qualified experts who visually inspect Giemsa slides, the quantification of parasitemia can vary several-fold to several tens-fold due to human error. Based on our observations at the clinical sites, when three users examined a single Giemsa thin slide using microscopy, the parasitemia counts ranged from 1,836 to 5,873 per microliter (19). This indicates that parasitemia levels can vary within the same slide depending on where the examiner reads. A similar situation can occur with miLab MAL slides if users observe the slides manually rather than automatically screening them. However, miLab has the advantage of automatically screening designated areas and using AI algorithms to preferentially select normal red blood cells, allowing users to observe a large area evenly with less effort.

Nonetheless, there is potential for bias depending on which areas miLab prioritizes for screening. This bias can be minimized by screening a larger number of red blood cells, thereby lowering the sampling ratio. Given that there are approximately 5,000,000 red blood cells per microliter of whole blood, screening 200,000 red blood cells equates to sampling about 1/25th of the loading volume. Therefore, to achieve more accurate quantification, it may be advisable to screen more than 500,000 red blood cells, which would likely yield even more precise quantitative values.

Despite studies that suggest that miLab may be more accurate, there were several limitations in assessing parasitemia by microscopy. Parasitemia in our study was calculated by counting approximately 1,000 erythrocytes. The World Health Organization standard, however, suggests that parasitemia counts be made per 200–500 leukocytes (20). Second, parasitemia was assessed with only one medical laboratory scientist instead of several experts. Third, the number of positive cases was limited which may have led to greater biases. Additional studies will be performed to address these limitations and to assess the accuracy of each method.

Timed studies have shown that it takes at least 30 minutes to view 100,000 erythrocytes within a thin film smear, using standard microscopy (20). Based upon this calculation, it would take a technologist more than an hour to look at about 200,000 erythrocytes. For low parasitemia samples, microscopists sometimes need to check more than 100,000 erythrocytes to get accurate parasitemia percentages or confirm negative results. During this study, it took an average of 18 minutes to check 200,000 erythrocytes using miLab MAL. The operator spent less than a minute loading the blood sample on the cartridge; the process was performed automatically. The miLab MAL instrument, therefore, not only reduced overall analysis time while providing more accurate results but also eliminated the need for microscopists to spend long hours peering into the microscope to screen negative samples.

The miLab MAL correctly identified *P. falciparum* species in 11/11 cases compared to the standard microscopic method. Based on the study’s findings, at minimum, the miLab MAL can be used to screen out *P. falciparum* samples reliably. Due to the limited number of positive samples (*n* = 11) and the lack of non*-P*. *falciparum* samples, further studies in a malaria-endemic area are necessary to assess the reliability of speciation and parasitemia. The appropriate diagnostic or clinical cut-off for each diagnostic laboratory could be verified if sufficient positive samples are obtained. If these studies are successful, this technology has the potential to be effectively utilized in laboratories performing malaria screening and speciation, as well as to be applied in malaria case monitoring and surveillance in malaria-endemic areas.
